# Identification and Characterization of Microsatellite *Loci* in Maqui (*Aristotelia chilensis* [Molina] Stunz) Using Next-Generation Sequencing (NGS)

**DOI:** 10.1371/journal.pone.0159825

**Published:** 2016-07-26

**Authors:** Adriana Bastías, Francisco Correa, Pamela Rojas, Rubén Almada, Carlos Muñoz, Boris Sagredo

**Affiliations:** 1 Instituto de Investigaciones Agropecuarias (INIA) CRI Rayentué, Av. Salamanca s/n, Sector Los Choapinos, Rengo, Chile; 2 Facultad de Ingeniería, Universidad de Talca, Avenida Lircay s/n, Talca; 3 Centro de Estudios Avanzados en Fruticultura (CEAF), Av. Salamanca s/n, Sector Los Choapinos, Rengo, Chile; 4 Facultad de Ciencias Agronómicas, Universidad de Chile, Avenida Santa Rosa N° 11315, La Pintana, Santiago de Chile, Chile; Wuhan Botanical Garden of Chinese Academy of Sciences, CHINA

## Abstract

Maqui (*Aristotelia chilensis* [Molina] Stunz) is a small dioecious tree native to South America with edible fruit characterized by very high antioxidant capacity and anthocyanin content. To preserve maqui as a genetic resource it is essential to study its genetic diversity. However, the complete genome is unknown and only a few gene sequences are available in databases. Simple sequence repeats (SSR) markers, which are neutral, co-dominant, reproducible and highly variable, are desirable to support genetic studies in maqui populations. By means of identification and characterization of microsatellite loci from a maqui genotype, using 454 sequencing technology, we develop a set of SSR for this species. Obtaining a total of 165,043 shotgun genome sequences, with an average read length of 387 bases, we covered 64 Mb of the maqui genome. Reads were assembled into 4,832 contigs, while 98,546 reads remained as singletons, generating a total of 103,378 consensus genomic sequences. A total of 24,494 SSR maqui markers were identified. Of them, 15,950 SSR maqui markers were classified as perfects. The most common SSR motifs were dinucleotide (31%), followed by tetranucleotide (26%) and trinucleotide motifs (24%). The motif AG/CT (28.4%) was the most abundant, while the motif AC (89 bp) was the largest. Eleven polymorphic SSRs were selected and used to analyze a population of 40 maqui genotypes. Polymorphism information content (PIC) ranged from 0.117 to 0.82, with an average of 0.58. Non-significant groups were observed in the maqui population, showing a panmictic genetic structure. In addition, we also predicted 11150 putative genes and 3 microRNAs (miRNAs) in maqui sequences. This results, including partial sequences of genes, some miRNAs and SSR markers from high throughput next generation sequencing (NGS) of maqui genomic DNA, constitute the first platform to undertake genetic and molecular studies of this important species.

## Introduction

Maqui (*Aristotelia chilensis* [Molina] Stunz) is a small dioecious tree native to South America that belongs to the Elaeocarpaceae family. It ranges from 3 to 4 m in height and grows in altitudes of up to 2,500 m.a.s.l. Maqui grows best in rich moist topsoil on hillsides or at forest edges. It is a pioneer species that colonizes newly grazed land, forming monospecific associations [1]. Maqui is an evergreen plant with serrated lanceolate leaves with a leathery texture and a reddish petiole. The fruit is a shiny black berry, 3–5 mm in diameter, which is used as food, dye and medicinal remedy [1–3].

Maqui fruit has analgesic, anti-inflammatory and antioxidant properties [4–6] and is characterized by high levels of antioxidant activity due to elevated anthocyanin and polyphenol content [4,7–9]. Maqui extracts have applications as food additives and nutraceutics because of the plant’s anthocyanin and polyglycosides content [10,11]. The fruit is collected from wild plants and overharvesting is threatening wild populations of *A*. *chilensis*. To preserve maqui as a genetic resource it is essential to study the genetics of this species. A better understanding of the genetic relationships among individuals and populations is needed for effective planning to preserve wild populations, as well as to estimate the genetic potential of traits with economic interest to breeding programs.

The genetic diversity of a population allows it to respond and adapt to environmental changes [12]. At the molecular level, genetic diversity in the form of variations in nucleotides, genes and genomes can be evaluated among different accessions of the same population (intraspecific) or among populations (interspecific) [13]. Genetic variation of a species is the fundamental basis of the evolution, adaptation and development of crop varieties [14]. Genetic variation emerges from the differences in DNA sequences (insertion, deletion, duplication or inversion). These differences can be functional, causing changes in metabolic or phenotypic characters, or neutral [15].

DNA molecular markers are powerful tools for analyzing genetic diversity. They are based on DNA sequence polymorphisms [16]. DNA molecular markers show Mendelian inheritance, ensure wide coverage of the genome and are neutral to environmental factors and stages of development as they are not affected by other genes or factors [15,17]. According to the development of technologies in molecular biology, DNA markers can be divided into three broad categories: first, second and third generation markers.

First generation markers are based on DNA-DNA hybridization, such as restriction fragment length polymorphism (RFLP). Second generation markers are based on DNA fragment amplification by PCR such as cleaved amplified polymorphic sequences (CAPs), amplified fragment length polymorphism (AFLP), sequence-characterized amplified regions (SCARs) and simple sequence repeats (SSRs), among others. Finally, third generation markers like single nucleotide polymorphisms (SNP) depend on a meaningful analysis of next-generation sequencing (NGS) data. Moreover, there are genome-scanning platforms based on array technology, such as expressed sequence tags (EST) and diversity arrays technology (DArT) that facilitate genome analysis [13,16,17].

The use of first generation markers has been drastically mainly because of limits on the number of samples that can be handled. At the same time, the high costs of third generation markers like SNPs and their derivative platform of arrays restricts their use in large-scale genotyping. In contrast, second generation PCR-based markers like SSRs are considered highly cost/effectivity [13,16].

Microsatellites, namely simple sequence repeats (SSRs), short tandem repetitions (STRs) and short sequences length polymorphisms (SSLPs), are short (1–6 bp) tandemly repeated DNA sequences [18]. SSRs are neutral, co-dominant, reproducible and highly variable molecular markers [19]. SSRs are found mainly in noncoding DNA regions whose origins and functions are unclear [20,21]. Microsatellites may generate genetic variation in the genome and influence transcriptional activity in promoter regions [22].

Based on the type of repeat sequence, microsatellites can be classified into three categories, perfect, imperfect and compound/composite, as defined by Weber (1990) [23]. However, other authors have defined distinct categories [21,24]. Perfect microsatellites are uninterrupted series of a repeat unit, e.g. (AT)_15_, while imperfect microsatellite sequences differ from perfect ones by the presence of one to three base interruptions in the run of tandem repeats, e.g. (AT)_10_C(AT)_8_. Finally, the sequence of a compound or composite microsatellite contains two adjacent distinctive sequence-repeats, e.g. (AT)7(TG)10. In general, the degree of polymorphism increases with the total length of the SSR. Longer and perfect SSR loci are known to exhibit greater allelic variability [25,26]. Significantly higher levels of genetic variation are found in perfect SSRs than in imperfect SSRs [27]. SSR markers have been used in many research areas such as linkage mapping [28], genetic diversity [29], phylogenetic analysis [30], genotype identification [31] and comparative genomic research [32].

SSRs have to be isolated and characterized for each species. Three general methods have been described for SSR isolation [13]: (i) the standard method, where a library is developed of genomic DNA, cDNA and PCR fragments [33], (ii) the automated method, where SSR sequences are searched in sequence databases and (iii) next generation sequencing (NGS), where the total or partial genome is sequenced using massive sequencing [34]. New massive sequencing platforms have enabled SSR sequencing from DNA or RNA sequencing. With these technologies it is not necessary to create a library as large numbers of sequences are rapidly produced. At the same time, the costs involved are decreasing [35–39].

In our work, we used high throughput next generation sequencing (NGS) to identify microsatellite or simple sequence repeat (SSR) markers from the maqui (*A*. *chilensis*) genome. In addition, partial sequences of genes and some regulator factors of maqui are described. This will constitute the first public platform to facilitate genetic and molecular studies of this promising species.

## Materials and Methods

### Plant Material

**Fig 1:**
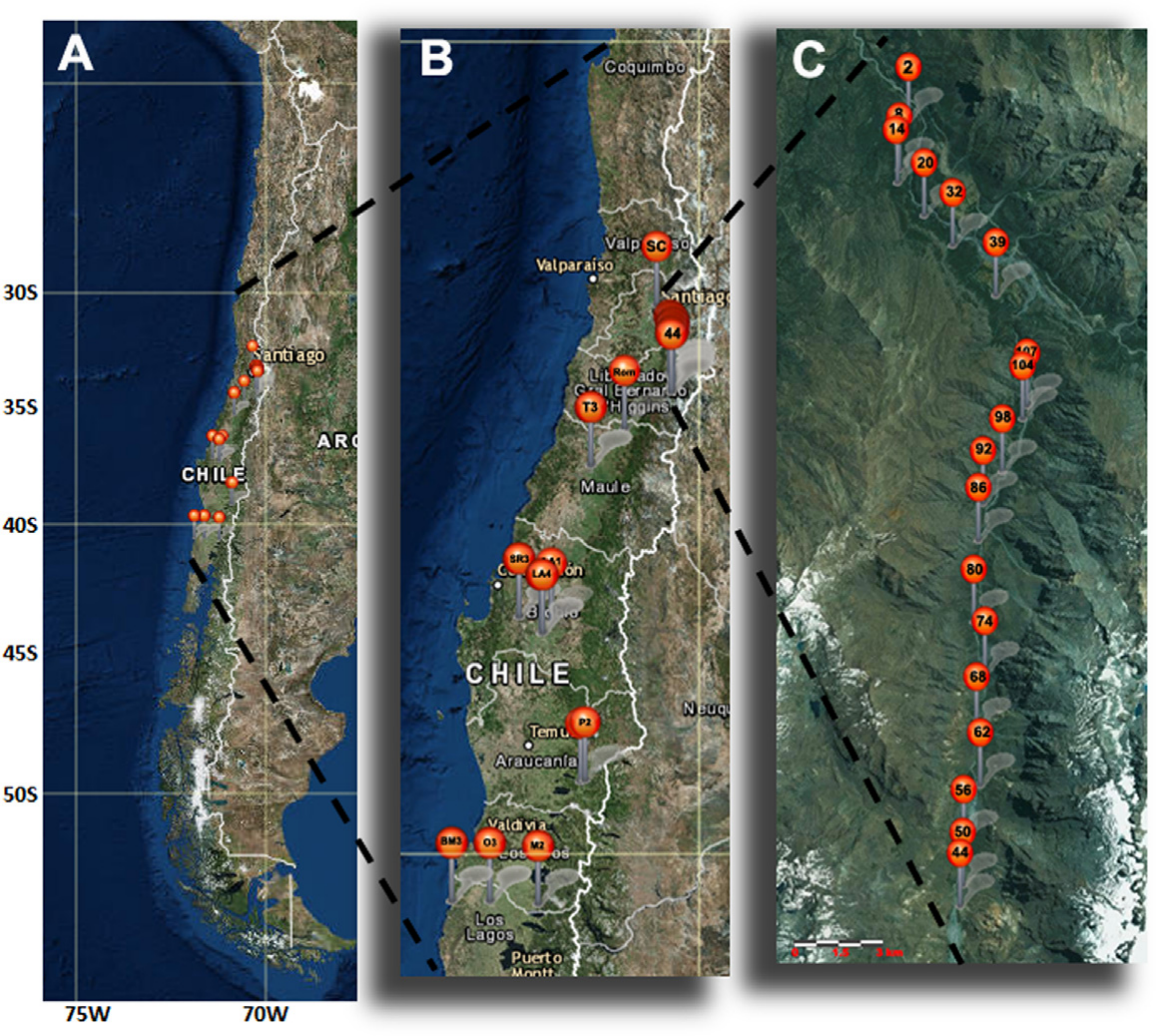
Sampling of maqui (*A*. *chilensis*) in Chile. Samples of maqui were collected from Santiago (33°25'0"S 70°39'0"W) to Los Lagos (40°34'57.41”S 73°43'55.80”W) of Chile. A is an aerial map of Chile. B is an enlargement of the sampled area, and C corresponds to sampling at the Río Los Cipreses National Reserve (O’Higgins Region, Chile). The original pictures were taken from USGS The National Map, http://viewer.nationalmap.gov/ (Coordinates of sampling are described in S1 Table).

Young maqui (*A*. *chilensis*) leaves were collected at the Río Los Cipreses National Reserve (O’Higgins Region, Chile) and other locations in Chile (Fig 1, S1 Table). Samples were frozen in liquid nitrogen and stored at −80°C until DNA extraction and subsequent analysis.

### DNA sequencing

Genomic DNA (approximately 100 mg) was extracted from leaves of one maqui plant (*A*. *chilensis*) named T1S (Fig 1, S1 Table) with the DNeasy Plant Mini Kit (Qiagen Inc., Valencia, CA, USA) following the manufacturer’s protocols. DNA quality and quantity were checked by agarose gel electrophoresis and spectrophotometric measurement of UV absorption at wavelengths of 260 and 280 nm and absorbance ratios of 260/280 and 260/230 using an Infinitive M200Pro Nanoquant (Tecan Group US, Inc., Morrisville, NC, USA).

The DNA was subjected to shotgun pyrosequencing (1/8th run) on the Roche 454 Genome Sequencer FLX at Macrogen Inc.(Seoul, Korea) using GS-FLX Titanium reagents (Roche Applied Science), as has been described by Margulies et al. (2005) [40]. GS FLX data processing was performed using the Roche GS FLX software (v 2.9). The obtained nucleotide sequence reads were assembled with GS De Novo Assembler (v 2.9) software.

### SSR detection and primer design

We only analyzed perfect SSRs. The contig and singleton sequences obtained in FASTA files were screened for di-, tri-, tetra-, penta-, hexa-, hepta- and octanucleotide repeats, and only dinucleotide repeats with lengths of ≥ 6, trinucleotide repeats with lengths of ≥ 4, and tetranucleotide to octanucleotide repeats with lengths of ≥ 3 were accepted using MIcroSAtellite identification software [41]. The program allows for direct primer design using PRIMER 3 [42] by searching for microsatellite repeats and primer annealing sites in the flanking regions.

### SSR Validation

Primers were successfully designed and synthetized for a total of 50 SSRs (at least three repeats) from sequencing data. A subset of DNA of ten individual maqui plants was prescreened for the quality of amplified SSRs by capillary electrophoresis on a fragment analyzer (Advanced Analytical Technologies, USA). We selected the polymorphic SSR loci with scorable polymorphic bands. The remaining loci that showed no amplification, multibanding patterns, or monomorphic bands were discarded. Finally, to characterize the confirmed loci, a total of 40 maqui (*A*. *chilensis*) individuals were sampled from the Rio Los Cipreses National Reserve and other locations in Chile (Fig 1, S1 Table).

The genomic DNA was extracted from the leaves of each sample using the DNeasy Plant Mini Kit (Qiagen Inc., Valencia, CA, USA) following the manufacturer’s instructions. The polymerase chain reaction (PCR) was carried out in a final volume of 25 μl containing 20 ng of genomic DNA, 2.5 μl 10 x Master mix (Stratagene Paq5000 DNA Polymerase buffer; Agilent Technologies, Santa Clara, CA, USA), 0.4 μl 10 mM dNTPs, 0.4 μl 10 uM of each forward and reverse primer, 1 U of Taq polymerase (Stratagene Paq5000 DNA Polymerase; Agilent Technologies, Santa Clara, CA, USA) and distilled nuclease-free water up to a final volume of 25 μl/tube with the following temperature profile: an initial 5 min at 95°C, 35 cycles of 45 s at 95°C, 80 s at an annealing temperature in accordance with the primers (S2 Table), 45 s at 72°C, and finally, 5 min at 72°C. PCRs were performed using a Swift Maxi Thermal Cycler model MX-BLC7 (Esco, Hatboro, PA, USA). The amplification products were separated by capillary electrophoresis using a Fragment Analyzer™ 12-capillary Automated CE System (Advanced analytical Technologies, Ames, IA, USA) using DNF-900 double-stranded DNA Reagent Kit (Advanced Analytical Technologies, Ames, IA, USA). The gel, inlet buffer, capillary conditioning solution, 35bp/500 bp marker and instrument were prepared according to the manufacturer’s instructions. To prepare the sample plate, 4 μl of each DNA sample and 22 μl of 1X TE dilution buffer were placed in the respective wells of the sample plate and first vortexed at 3000 rpm for 2 min and then centrifuged. The sample plate was run immediately after being prepared. To run the samples, the plate was placed in one sample plate tray of the Fragment Analyzer™. The DNF-900 dsDNA Reagent Kit experimental method (12-Capillary, 50–80 array) was loaded and run.

### Data analysis of putative alleles

After capillary electrophoresis separation, the data was opened and processed using the software PROSize® 2.0 version 1.3.1.1 (Advanced Analytical Technologies, Inc., Ames, IA, USA). The data were normalized to the lowest (35 bp) and highest markers (500 bp), and calibrated to the 75–400 bp Range DNA ladder. A peak was considered a putative allele when the percentage of the area under the curve was more than 10%, with a maximum of two counted alleles per individual.

A double-entry matrix was developed with all counted alleles. Simple sequence-repeat alleles were scored as present (1) or absent (0). An index of similarity among pairs was developed, as well as a neighbor-joining method to generate a dendrogram. A bootstrap of 1000 was included to evaluate the robustness of the distribution tree. All analyses were performed with DARwin 6.0.012 software [43].

### Polymorphism information content (PIC)

The PIC values of all the polymorphic SSR markers were calculated as follows: $PIC=1-\sum_{i=1}^{k}P_{i}^{2}$, where k is the total number of alleles detected for a given marker locus and *Pi* is the frequency of the i*th* allele in the set of investigated genotypes [44].

### Putative maqui gene prediction

Putative genes were predicted with AUGUSTUS software [45], analyzing contig and singleton genomic sequences from maqui. The program is based on a hidden Markov model and is used for the *ab initio* prediction of protein coding genes in eukaryotic genomes. *Arabidopsis thaliana* (L.), Heynh. was used as the model organism. BLAST2GO was then used to functionally annotate potential coding sequences or predicted genes [46]. This research tool was designed to allow consistent gene annotation and Gene Ontology (GO) based data mining of sequence data for which GO annotation is not yet available. The sequences were analyzed with Blastx tool against a base of customized local database with sequences of different plant species, giving a description of the best hit of mapped sequence, GO terms, enzyme commission (EC) number and InterPro partners. The sequences were identified in a non-redundant database with a personalized e-value cutoff of E -6.

### Predicting microRNAs

Potential microRNAs were computationally predicted using a hidden hierarchical Markov model (HHMMIR) [47], which allows *de novo* prediction of microRNAs using hidden Markov hierarchical models. These results were validated using miPred [48] and miRdup [49] tools, with which respectively we distinguished the real pre-miRNAs from other hairpin sequences with similar stem-loops and predicted the location of microRNAs in their pre-miRNA. Plant miRNAs described in the miRbase database [50] were used as models.

## Results

### SSR detection from NGS sequence analysis

SSR markers can be developed using next generation sequencing for species like maqui (*Aristotelia chilensis*), which lack genomic data [35]. In our study, the genomic DNA of maqui was partially sequenced by the *de novo* shotgun 454 pyrosequencing. A total of 165043 bp pair-end reads were obtained with an average read length of 408 bp, covering 64 Mb of the maqui genome (Table 1).

**Table 1:** Number of maqui (*A*. *chilensis*) 454 sequences before and after assembly.

Item	Total Number
Reads generated	165043
Total bases	64018929
Average read length	387.893
Assembled sequence	43660
Contigs sequence	4832
Singleton sequence	98546
% GC	38.94

Reads were assembled into 4832 contigs, while 98546 reads remained as singletons, generating a total of 103378 consensus genomic sequences (Tables 1 and 2). About 80% of contigs were assembled with fewer than 30 reads. The average GC content of genomic maqui DNA was 38.94%.

**Table 2:** Results of microsatellite (SSRs) search from maqui (*A*. *chilensis*) using MIcroSAtellite identification tool.

Category	Numbers
Total number of sequences examined	103378
Total size of examined sequences (bp)	40317.005
Total number of identified SSRs	24494
Total number of identified perfect SSRs	15950
Number of perfect SSR containing sequences	13531
Number of sequences containing more than 1 perfect SSR	1963

#### Identifying maqui SSR in genomic sequences

With the goal of determining a set of genetic markers in maqui (*A*. *chilensis*), contigs and singleton sequences were surveyed for the presence of SSRs by means of the MIcroSAtellite (MISA) tool [41]. The total of identified SSR sequences was 24494 (Table 2, S3 Table). S3 Table shows the putative SSR markers and their possible primer pairs. However, only perfect SSRs were considered in the following analysis, with a repeat motif size range of 2 to 8 bp and a length of >12 bp. This includes dinucleotide repeats ≥ 6, trinucleotide repeats ≥4, and tetra-, penta-, hexa-, hepta- and octanucleotide repeats ≥3. All the mono-nucleotide repeat SSRs were excluded from this analysis. A total of 15950 SSRs were identified, which were contained in 13531 contigs and singleton sequences (Tables 2 and 3).

**Table 3:** Characterization of SSRs in genomic sequences of maqui (*A*. *chilensis*) generated by 454 sequencing.

Item	50–100 bp	101–200 bp	201–300 bp	301–400 bp	>400 bp	Total
Contig sequences	20	1164	829	687	2132	4832
Singleton sequences	2435	7584	12625	24194	51708	98546
Sequences containig SSR	240	869	1410	3040	7972	13531

#### Distribution of SSR motif lengths and types and repeat numbers

**Fig 2:**
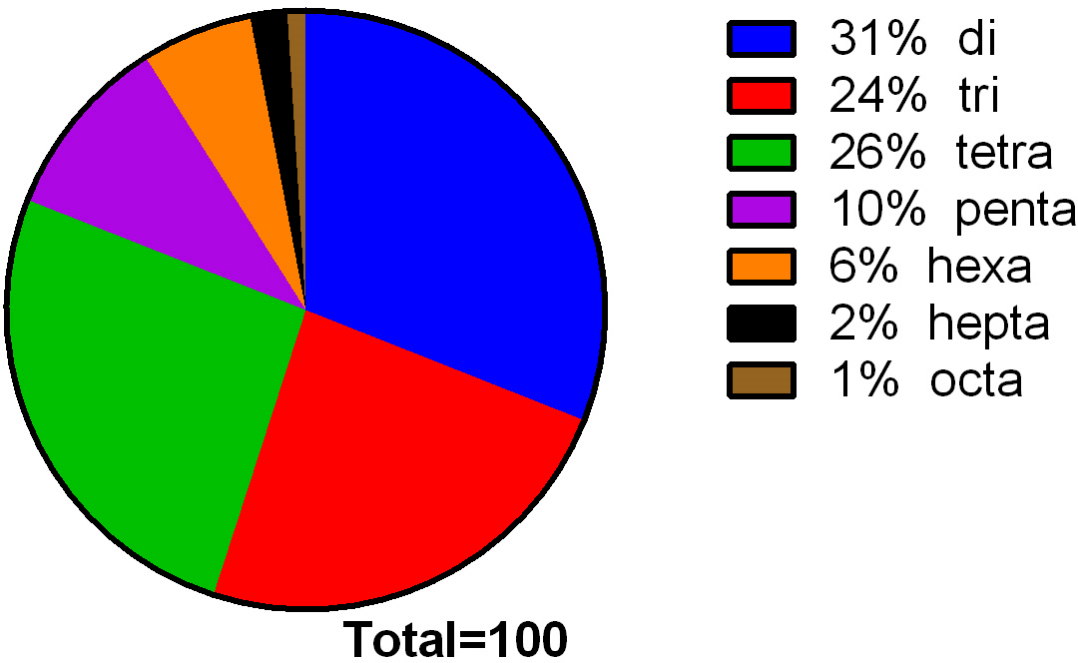
Frequency distribution of SSR loci of maqui (*A*. *chilensis*) by motif length. The graph is based on a total of 15950 SSR markers detected in non-redundant genomic maqui DNA. Di, tri, tetra, penta, hexa, hepta and octa refer to dinucleotides, trinucleotides, tetranucleotides, pentanucleotides, hexanucleotides, heptanucleotides and octanucleotides, respectively.

**Fig 3:**
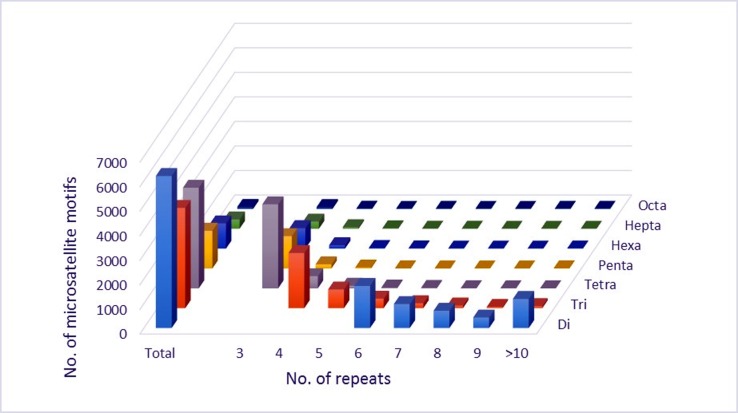
Distribution of SSR from maqui (*A*. *chilensis*) with Di- to Octa-nucleotides by repeat numbers. The graph is based on a total of 15950 SSRs detected in non-redundant genomic maqui DNA. Di, tri, tetra, penta, hexa, hepta and octa refer to dinucleotides, trinucleotides, tetranucleotides, pentanucletides, hexanucleotides, heptanucleotides and octanucleotides, respectively.

We examined the distribution of maqui microsatellites with regard to motif length and type and the number of repeats (Figs 2 and 3, S4 and S5 Tables).

In regard to SSR motif length, dinucleotide (31.42% of total) and tetranucleotide (25.58% of total) repeats were the most abundant. While, hepta- and octa-nucleotide repeats were the least common, representing together less than 3% of total SSRs (Fig 2, S4 Table). SSR frequency decreased as the motif size increased. The mean number of dinucleotide repeats was 8.48, almost twice as many as trinucleotides and three times as many as tetra- to octanucleotides (S4 Table).

Regarding the distribution of identified SSRs according to the motif type and number of repeats, three-repeat tetranucleotides were the most abundant SSR motif (3424), which was 21.47% of identified perfect SSRs, followed by four-repeat trinucleotides (14.07%) (S4 Table). Di- and trinucleotides with fewer than four repeats were not included in this analysis. The largest SSR identified was the AC dinucleotide with 89 bp, followed by the AGT trinucleotide with 27 bp (Table 4).The most common SSR was AG/CT, with 28.4% of total perfect SSRs, followed by ATT/AAT (Table 4).

**Table 4:** Summary of the frequency of SSRs from maqui (*A*. *chilensis*) with different numbers of tandem repeats.

Motif length	SSR (Major size [bp])	More frequently (%)
Di	AC (89)	AG/CT (28.4)
Tri	AGT(27)	ATT/AAT (15.8)
Tetra	AAGA(9)	AAAT/ATTT (12.6)
Penta	CCTAA(17)	ATTTT/AAAAT (10.2)
Hexa	ACCCTA (26)	TTTTTA/TAAAAA (11.8)
Hepta	TAACCCG (7)	TTTTTTA/TAAAAAA(11.5)
Octa	TAAACCCG (10)	TTTTTTTA/TAAAAAAA (13)

#### Development of SSR genomic markers

Primer pairs were designed and synthesized for a total of 50 non-redundant sequence SSRs (S2 Table). All 50 primer pairs were screened for amplification of DNA in standard conditions from a mix of ten maqui genotypes. This analysis identified a set of 44 markers (90%) that amplified expected size amplicons. However, only a subset of 11 SSR markers showed scorable polymorphic bands (Table 5). The remaining 39 SSR loci showed no amplification, multibanding patterns, or monomorphic bands. Thus, with these 11 SSR markers we genotyped 40 maqui accessions, including 17 from Río Los Cipreses National Reserve and 23 other locations in Chile (Fig 1, S1 Table).

**Table 5:** Characteristics of 11 nuclear microsatellite loci developed for maqui (*A*. *chilensis*). For each locus, the forward and reverse primer sequences, repeat motif and annealing temperature when run individually (Ta) are shown.

ID	Primers forward/reverse (5 ′ – 3 ′)	Repeat	T a (°C)
M4	ACATCCCCTTAAAAGAACCCCT / ACAAATGTGCTGGTCGTCAT	(TATG)_4_	58
M9	TAACAGCTTGCGATGCCATG / AGCCGTATAGGACCACATGA	(TTCT)_3_	58
M15	TGAGCATCAACTCACTCAAATG / GCTGTAAATCTGCTTGCCTGTA	(CATG)_3_	59
M18	TTACCACACAAAACGTATCCCA / CACTATCGAACAAAGGGAAAGC	(TTCT)_3_	60
M31	AAAAGTAGGAGGCAAGGATTGA / CTAGCGAAGGTTCCCATGATAC	(TTTA)_3_	59
M32	TGTCTTGTTTAGGCATTTGGTG / TACGAAGATTTCCCTTCTTTGC	(TTTC)_3_	59
M33	GAAAGGGTCACGGATCATTCTA / AATCACCCAATAAGGAAGCTCA	(AAAT)_3_	59
M34	GCAGAAGTCAAAGAAAAGCCAT / CTCAGCCCACACAATAGTAACG	(TTTA)_3_	59
M35	AGCCATCACTTGGAATGGTAAT / TCAGAAAACGATAGATGCCCTT	(AAAC)_3_	59
M37	CCTCCGGTACTTCACTTTATCG / CCAGGAGAAAAGCATCGAGT	(GAAT)_3_	59
M45	AACGAAGAGCAAACAGTAGGAA / TCGTAAACCCAGATGTCTTAGG	(TTTA)_3_	58

Allelic data obtained from 40 genotypes were used to calculate the polymorphism information content (PIC) of each SSR marker, which ranged from 0.117 to 0.820, with an average of 0.584 (Table 6).

**Table 6:** Characterization of SSR markers on 40 maqui (*A*. *chilensis*) genotypes from Chile.

Marker	Alleles (N°)	Size range (pb)	PIC
M4	7	117–170	0.588
M9	5	256–320	0.567
M15	5	219–235	0.442
M18	8	117–193	0.713
M31	3	138–158	0.152
M32	4	177–185	0.117
M33	5	124–132	0.653
M34	10	147–195	0.820
M35	12	107–191	0.799
M37	11	148–192	0.802
M45	7	282–298	0.772

### Phylogenetic relationship among maqui genotypes from Río Los Cipreses Reserve and other locations in Chile

**Fig 4:**
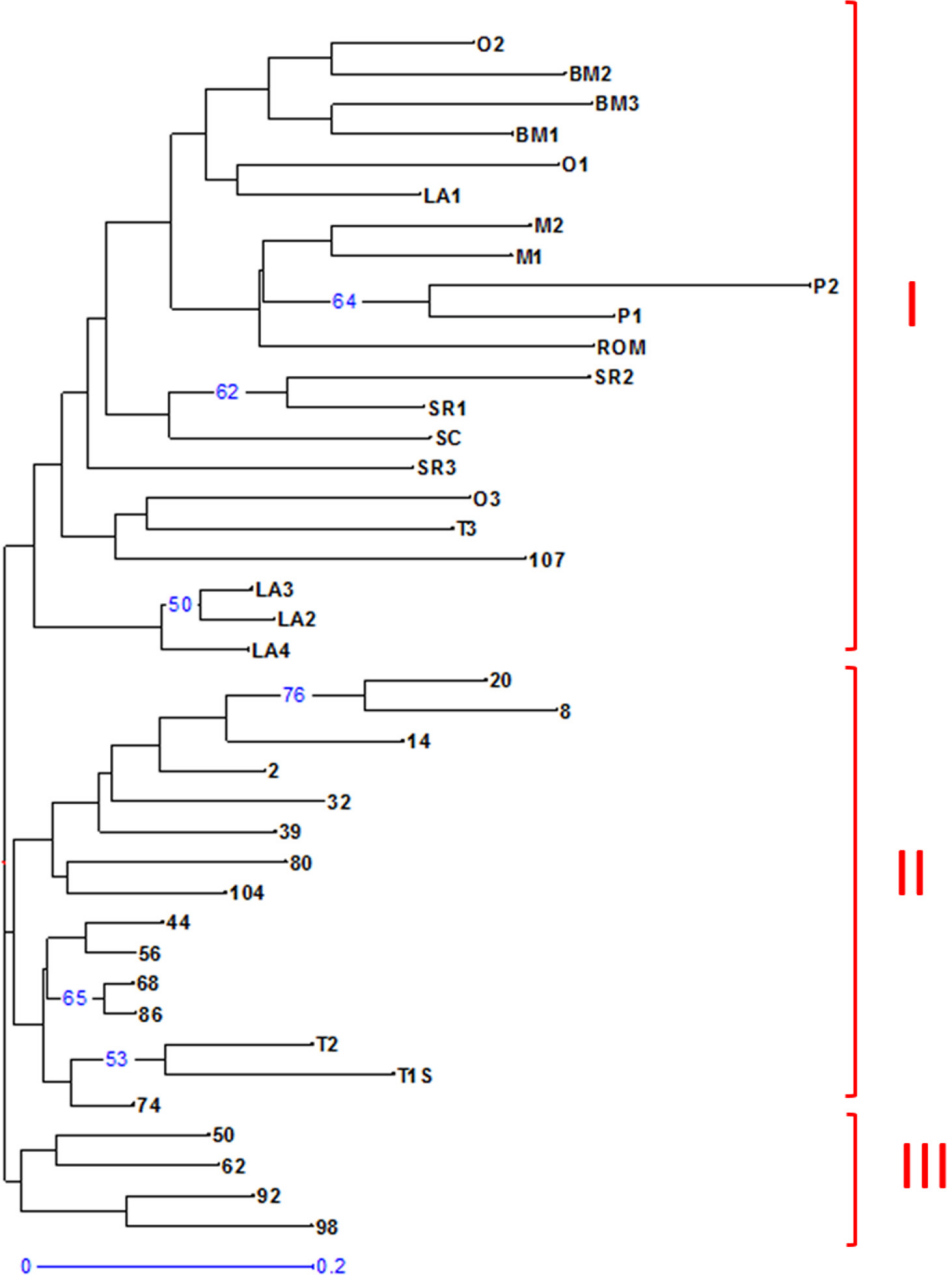
Neighbor-joining tree of maqui (*A*. *chilensis*) genotypes. Forty maqui genotypes were analyzed based on 11 SSR locus variations using DARwin V6. 0.012 software and bootstrap analysis of 1000. Numbers indicate genotypes from Río Los Cipreses National Reserve and letters indicate other locations in Chile where the samples were collected, T: Talca, SC: San Cristóbal Hill, ROM: Romeral, SR: San Rosendo, O: Osorno, M: Mantilhue, P: Pucón, BM: Bahía Mansa and LA: Los Ángeles. I, II and III, correspond to branches I, II and III, respectively.

Fig 4 shows the phylogenetic tree of the 40 maqui genotypes. Three main branches were observed, but they were not supported by significant bootstrap values, suggesting a panmictic structure of maqui. However the 40 genotypes tend to be grouped according to their geographic location. One branch is composed of samples from San Cristobal hill (Metropolitana Region) to Osorno (Los Lagos Region), including a sample from Río Los Cipreses National Reserve. Another branch is composed of two samples from Talca (Maule Region) and the majority of samples from Río Los Cipreses National Reserve with the exception of samples 50, 62 92 and 98, which tend to be grouped in other branch.

### Prediction of putative maqui genes

The contig and singleton maqui genomic sequences were analyzed by AUGUSTUS software [45] to predict 11150 putative genes using *A*. *thaliana* as a model organism (S6 Table). For functional annotation, the potential coding regions were analyzed by BLAST2GO [46], leading to consistent gene annotations, gene names, gene products and Gene Ontology (GO) numbers. The functional search identified 6115 homologous sequences on the non-redundant custom database.

**Fig 5:**
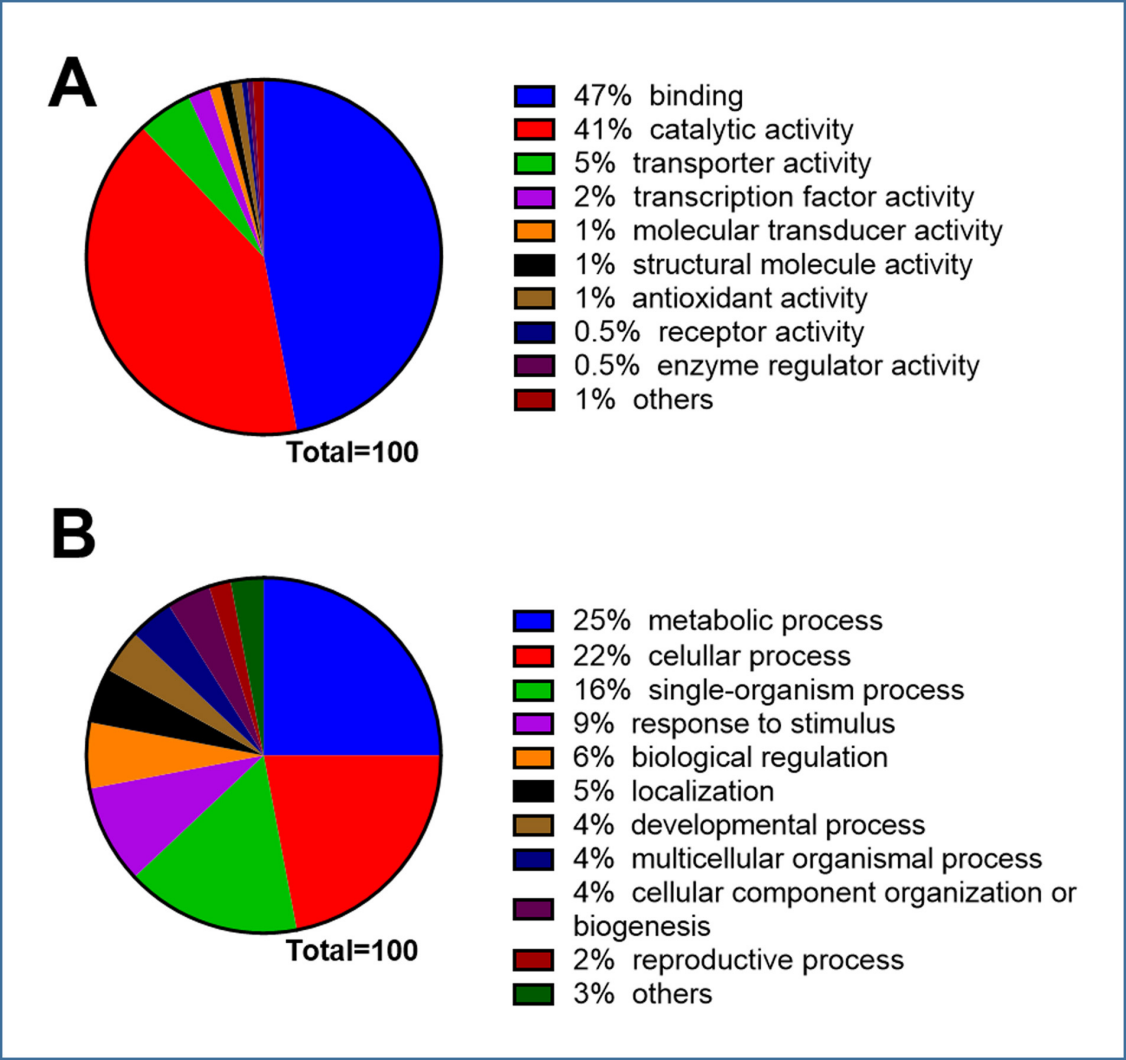
Gene Ontology classification of the predicted maqui (*A*. *chilensis*) ORFs. The classification was predicted according to molecular function (A) and biological process (B) using BLAST2GO with an E-6 cutoff.

Gene Ontology allows for categorizing gene products according to three ontologies: molecular function, biological process and cellular component. The search for homologous sequences found 6115 sequences on the non-redundant database. Under molecular function ontology, a large proportion of genes were assigned to two categories: binding (47%) and catalytic activities (41%) (Fig 5A), while under biological process ontology the majority of genes were categorized into metabolic (25%), cellular (21%) and single organism processes (Fig 5B).

### Prediction of microRNAs in maqui (*A*. *chilensis*)

MicroRNAs are short RNA species that act as key post-transcriptional regulators [51]. We used the HHMMIR approach [47] to predict 9229 pre-miRNAs, which were subsequently reduced to 1887 putative candidate miRNAs using miPred and miRdup tools [48–49]. We then searched for regions of local similarity among candidate miRNA sequences using the Basic Local Alignment Search Tool (BLAST) against different plant species to find conserved putative miRNAs.

Three candidate miRNAs were homologous with sequences in miRbase databases, namely miR156, miR812 and miR5149, whose target genes are squamosa-promoters like protein (SPL), ATP binding protein and RPS2 protein, respectively [52–54] (Table 7).

**Table 7:** Results from homology search of microRNA candidates from maqui (*A*. *chilensis*) against the microRNA database.

Read name	miRNA family	MFE (kcal/mol)		Target	Reference
IYJ1KEP07ICDQ4	miR156		-16.40	Squamosa-promoter Binding Protein (SBP)-like transcription factors	Rajagopalan et al., 2006
IYJ1KEP07IH1FS	miR812		-31.70	ATP binding protein, putative, expressed	Zhu et al., 2008
IYJ1KEP07IJFG2	miR5149		-19.00	Disease resistance protein RPS2, putative, expressed	Chen et al., 2011

## Discussion

This study provides the first set of nuclear microsatellite (SSR) loci for maqui (*A*. *chilensis*) by next generation sequencing (NGS). We used 454 pyrosequencing data to obtain a large number (15950) of potential microsatellite sequences. Using a population of 40 maqui genotypes collected mainly at Río Los Cipreses National Reserve (O’Higgins Region, Chile) we characterized 50 SSR. A subset of 11 polymorphic SSR loci that presented good scoring quality was selected for further genetic studies. These microsatellite loci will be useful to investigate genetic structure and gene flow and for developing maqui breeding strategies. This is the first time that part of maqui genome has been made known, which is important in order to learn more about this species with potential as an antioxidant, natural dye and food source [1–3].

Recent developments in sequencing technologies and bioinformatic analysis provide an unprecedented opportunity to discover SSR markers of high quality and effective cost/time in non-model organisms, like maqui, about which genomic information was lacking [35,37,55]. *De novo* 454 pyrosequencing covered around 64 Mb of the maqui genome, with 165043 reads and 64,018,929 bases (Table 2). An average of 38.94% of GC content of maqui genomic DNA was generated in this study, which is consistent with reports on GC content in other plant genomes like arabidopsis (36%), cucumber (32.3%) grape (34.4%), rice (43.6%) and potato (34.8%) [55,56].

We predicted 11150 putative maqui genes with this information and obtained partial sequences. Ontological analysis of molecular function showed a large proportion classified in binding (47%) and catalytic activities (41%) (Fig 5A), while the ontology of biological processes revealed large proportions in metabolic and cellular processes (Fig 5B). Although these results represent a partial sequencing of the maqui genome, they are the first global identification of maqui genes (S6 Table). Interesting, we also found 14 putative anthocyanidin-related genes from maqui sequences (S7 Table). Among them are putative structural genes like *phenylalanine ammonia-lyase* and *chalcone synthase* and some putative regulator genes.

We identified three microRNAs (Table 7), which are short RNA species derived from hairpin-forming miRNA precursors (pre-miRNA) and acting as key post-transcriptional regulators [51]. Most computational tools labeled as miRNA predictors are in fact pre-miRNA predictors and provide no information about the putative miRNA location in the pre-miRNA [47–49]. The identified miRNAs were miR156, miR812 and miR5149, which appear to be related to vegetative phase change, cold stress, and plant disease resistance, respectively [57–59].

Data from 454 sequencing were used to obtain a total of 25494 putative SSR of maqui, where 15950 were classified as perfect microsatellites (Table 3, S3 Table). We detected a density of 382.72 SSR markers per Mb (or 1 SSR each 2.61 kb), considering the total of putative SSR. A higher density of SSRs is described in plants as cucumber [55] but a less density is observed in pigeonpea, among others [60].

The identified microsatellites were genomic SSRs. The development of genic SSRs, SSRs from transcriptomic sequences, is limited to species for which sufficient sequence data are available [61]. Genomic SSRs are better than genic SSRs for fingerprinting or varietal identification studies because greater DNA sequence conservation in transcribed regions in genic SSRs results in less polymorphism, making them less useful than genomic SSRs for distinguishing closely related genotypes [62].

In this study, we analyzed the distribution and frequency of perfect microsatellites with a range of 2 to 8 SSRs and a length >12 bp. The perfect SSR markers are DNA sequences in which a fragment is repeated consecutively without interruption. We used only perfect SSRs because they have greater genetic variation than imperfect SSRs [27]. We considered dinucleotide repeats of ≥ 6, trinucleotide repeats of ≥4, and tetra-, penta-, hexa-, hepta- and octanucleotide repeats of ≥3 (Tables 3 and 4), while mono-nucleotide repeats were excluded from the analysis. The largest percentage (31%) of the 15950 perfect SSRs were dinucleotide sequences (Fig 2), as is the case with the European alder (*Alnus glutinosa* (L.) Gaertn.), cranberry (*Vaccinium macrocarpon* Ait.) mung bean (*Vigna radiata* (L.) Wilczek) and others [37,63–66]. The number of motifs of all types of SSRs decreased as the number of repeats increased. The SSR markers with two tandem repeats had the highest frequency (31, 42%), followed by SSR markers with four tandem repeats (25, 58%) and three tandem repeats (24, 10%) (Fig 2 and S4 Table).

The most common dinucleotide SSR is AG/CT, with a frequency of 28 (4%). With 89pb, AC is the longest SSR (Table 5). In general, AT-rich repeats prevail in dicot species, but not in monocots [55]. In our case, this result could be because is a partial sequencing of maqui genome. AT-rich repeats tend to predominate in trinucleotide SSRs in different plants [55], as was the case in our study, where ATT/AAT was the most common trinucleotide (Table 5).

Based on the identified SSR sequences, fifty pairs of primers were synthetized to evaluate polymorphic SSR loci. Once the polymorphic SSR loci were established, some were used to evaluate their variability in maqui genotypes. Based on 11 polymorphic SSR loci, the phylogenetic tree of the 40 maqui genotypes tend to be grouped into three branches, but they were not supported by significant bootstrap values. This is suggesting a panmictic genetic structure of maqui. This could be due to gene flow throughout the species’ growth distribution and seed dispersion by birds [67,68]. Two branches represent 90% of the samples. One branch is composed of samples from San Cristobal Hill (Metropolitana Region) to Osorno (Los Lagos Region). Another branch is composed by two samples from Talca (Maule Region) and the samples from Río Los Cipreses National Reserve, with the exception of samples number 50, 62, 92 and 98 (Fig 4). Because Río Los Cipreses National Reserve was previously managed as a farm (CONAF staff and A. Lara, Personal Communication) and it suffered big fires before to become a protected reserve, most maqui genotypes come from new seeds (dispersed by birds) from surrounding areas. This fact might explain the low differentiation of its maqui population with the rest of the country. Further studies with bigger sizes of populations and higher numbers of SSRs are necessary answer this type of questions.

Previously, a study differentiated four sampling sites/geographic regions using a fingerprinting approach with inter simple sequence repeats (ISSRs) [67]. However, some maqui genotypes were included in clusters not associated with their geographic origins. The ISSR multi-loci technique has certain disadvantages like the possible non-homology of fragments of similar size and reproducibility problems like RAPDs [69].

Genome-wide analysis of SSRs, coupled with information on their distribution in coding and non-coding regions, can provide insights into the role of SSRs in gene regulation and genome organization [55]. This will be easier to achieve with maqui once its genome has been elucidated.

In this study we used next generation sequencing to identify partial gene sequences, some miRNAs and a large set of SSR markers for first time in maqui (*A*. *chilensis*). This information is an important resource for genetic, genomic and evolutionary studies and will aid maqui conservation and breeding programs.

## Supporting Information

S1 Table List of genotypes from maqui (*A*. *chilensis*) used and their location (PDF)

S2 Table Characteristics of 50 putative nuclear microsatellite loci developed for maqui (*A*. *chilensis*). For each locus, the forward and reverse primer sequences, repeat motif and annealing temperature when run individually (Ta) are shown.
(PDF)

S3 Table Total identified SSR markers from maqui (*A*. *chilensis*) by MIcroSAtellite (MISA) tool with its respective primer pairs (XLSX)

S4 Table Frequency distribution of microsatellite loci of maqui (*A*. *chilensis*) by motif length (PDF)

S5 Table Distribution of identified SSRs from maqui (*A*. *chilensis*) using MISA software according to SSR motif type and repeat number (PDF)

S6 Table Putative genes found in partial sequences of maqui (*A*. *chilensis*) predicted by Blast2go software (XLSX)

S7 Table Putative anthocyanidin-related genes found in partial sequences of maqui (*A*. *chilensis*) predicted by Blast2go software (PDF)
