# Supplementary material for: Identification and Characterization of Microsatellite Loci in Maqui (Aristotelia chilensis [Molina] Stunz) Using Next-Generation Sequencing (NGS)
Source: PLoS One. 2016 Jul 26;11(7):e0159825. doi: 10.1371/journal.pone.0159825 (PMC4961369; doi:10.1371/journal.pone.0159825)
Supplement: S1 Table — (PDF) [file pone.0159825.s001.pdf]

S1 Table. List of genotypes from maqui (*A. chilensis*) used and their location

Accession ID	Latitude (S)	Longitude (W)	Chile Region
SC	33°25'0"	70°39'0"	Metropolitana
2	34°17'15.8"	70°26'46.4"	O'Higgins
8	34°18'07.1"	70°26'59"	O'Higgins
14	34°18'20.5"	70°27'01.2"	O'Higgins
20	34°18'54.6"	70°26'25.6"	O'Higgins
32	34°19'24.3"	70°25'47.0"	O'Higgins
39	34°20'16"	70°24'48.3"	O'Higgins
107	34°22'08.7"	70°24'06.4"	O'Higgins
104	34°22'22.7"	70°24'12.7"	O'Higgins
98	34°23'15.9"	70°24'39.6"	O'Higgins
92	34°23'49.0"	70°25'05.6"	O'Higgins
86	34°24'26.8"	70°25'12.0"	O'Higgins
80	34°25'50.7"	70°25'18.4"	O'Higgins
74	34°26'43.1"	70°25'03.9"	O'Higgins
68	34°27'40.8"	70°25'14.9"	O'Higgins
62	34°28'37.5"	70°25'10.0"	O'Higgins
56	34°29'35.3"	70°25'31.4"	O'Higgins
50	34°30'19.5"	70°25'32.6"	O'Higgins
44	34°30'40.3"	70°25'36.8"	O'Higgins
Rom	34°57'44.24"	71°7'55.18"	Maule
T1S	35°25'28.77"	71°37'56.6"	Maule
T2	35°25'28.6"	71°37'56.8"	Maule
T3	35°25'28.57"	71°37'56.93"	Maule
SR1	37°15'36.40"	72°43'30.85"	Bío-Bío
SR2	37°15'51.67"	72°43'26.26"	Bío-Bío
SR3	37°15'51.76"	72°43'3.81"	Bío-Bío
LA1	37°18'6.62"	72°14'1.70"	Bío-Bío
LA2	37°27'26.69"	72°23'27.71"	Bío-Bío
LA3	37°27'23.77"	72°22'30.38"	Bío-Bío
LA4	37°26'50.27"	72°22'22.10"	Bío-Bío
P1	39°12'5.88"	71°48'10.60"	Araucanía
P2	39°11'51.93"	71°43'51.82"	Araucanía
M1	40°37'17.09"	72°25'58.36"	Los Ríos
M2	40°37'15.23"	72°25'54.25"	Los Ríos
O1	40°35'22.97"	73° 9'49.62"	Los Lagos
O2	40°35'23.89"	73° 9'46.13"	Los Lagos
O3	40°35'24.47"	73° 9'50.55"	Los Lagos
BM1	40°35'4.74"	73°43'43.57"	Los Lagos
BM2	40°34'56.52"	73°43'57.29"	Los Lagos
BM3	40°34'57.41"	73°43'55.80"	Los Lagos
