# Supplementary material for: Identification and Characterization of Microsatellite Loci in Maqui (Aristotelia chilensis [Molina] Stunz) Using Next-Generation Sequencing (NGS)
Source: PLoS One. 2016 Jul 26;11(7):e0159825. doi: 10.1371/journal.pone.0159825 (PMC4961369; doi:10.1371/journal.pone.0159825)
Supplement: S2 Table — For each locus, the forward and reverse primer sequences, repeat motif and annealing temperature when run individually (Ta) are shown. (PDF) [file pone.0159825.s002.pdf]

S2 Table. Characteristics of 50 putative nuclear microsatellite loci developed for maqui (*A. chilensis*). For each locus, the forward and reverse primer sequences, repeat motif and annealing temperature when run individually (Ta) are shown.

ID	Primers forward/reverse (5 ' – 3 ')	Repeat	T a (°C)
M1	GGGTGAGGAAAATCGCAACT/AAAGCAAAATTACTCCGTTGAA	$(TTTA)_4$	55
M2	TGCACTTGAGTCTAAGCCGA/TCGGGGAAATGTTGCATCAC	$(ATTT)_4$	58
M3	TGAGTCTCCTTTCGCGTGAA/TCCTCTTCCTATGCAGCTCT	$(TGTT)_5$	57
M4	ACATCCCCTTAAAAGAACCCCT/ACAAATGTGCTGGTCGTCAT	$(TATG)_4$	58
M5	TGCAAGTCCTCTCAAGACAAAG/TCCGGATCCAAGAAACAGCT	$(ACTC)_4$	58
M6	AACGTCTGCCCTGATACACA/TTTTTTTTAATGACATTGCTTTGTG	$(AATA)_4$	56
M7	CATATGAACCTGTCAGTAACCT/GAGATGGGGTATAAGAAGAAATG	$(CTTG)_3$	58
M8	AAAGGGGATGAGAGGGTGAC/GTGGCTGACTTTCTTGCTGA	$(TGGT)_3$	58
M9	TAACAGCTTGCGATGCCATG/AGCCGTATAGGACCACATGA	$(TTCT)_3$	58
M10	GAGAGTGATGGAGGAAACTTGG/GCATCCCAGGCTATTGAAGT	$(TTTA)_3$	59
M11	AGCTCGTGAACCTTGATTCTTG/AAACAGAGAGGCAGAGAGATGG	$(TTTA)_3$	60
M12	ACCGAATATCGTAATCAACGGA/AGTTGGTGCTATGTCGAATGTG	$(ATCA)_3$	60
M13	GATGCAGATCATGGAAGAGCTA/CAGTCGAACTCCACCACAAAT	$(AACA)_3$	59
M14	GCCCAAGCACCCAAGTAT/TGGTAATGTGATGATGATGGTG	$(AAAT)_3$	58
M15	TGAGCATCAACTCACTCAAATG/GCTGTAAATCTGCTTGCCTGTA	$(CATG)_3$	59
M16	TAGGGCATACAACAATGGATCA/AGTCGGAAATAGAGCAGTGGC	$(GAAT)_3$	60
M17	GTTCTAGCTCTTCCAACCTTCG/AGCAATCCAGAAGCCGATATAC	$(CTTC)_3$	59
M18	TTACCACACAAAACGTATCCCA/CACTATCGAACAAAGGGAAAGC	$(TTCT)_3$	60
M19	TTACCACACAAAACGTATCCCA/CACTATCGAACAAAGGGAAAGC	$(TTCT)_3$	60
M20	CATGGCACACCTAGTTAGTTTATGA/CAGTGGTGGGGATGAGTTTT	$(ATTT)_3$	59
M21	CACCAATGAGCGTCTTGTCTC/ATTTAGGTATGCTTTTGGCCCT	$(TAAA)_3$	60
M22	ATATACCCCATTGCCAGCC/ACAATCGAGCAAAGAGAAGACC	$(TTCT)_3$	59
M23	CGGTCCAGTTGTTACTGATCCT/GCACCGAGTTAAATACATAAACACC	$(ATTT)_3$	60
M24	AGGGTGGTGTTAGGGATAATACTG/TGGCAAGTGAGTGAACAAAACT	$(TTTA)_3$	59
M25	TCACACACCAAACTCTATTGGC/TTTCCTTAGCTTCACGACCTTC	$(AGTT)_3$	60
M26	CACAGAGGATGGGGAGAGTTAT/TCAACCTAGATGTCCAGAAGATAGA	$(AAAG)_3$	58
M27	CGTTGTAATGGCACTTGTTTTG/TGACATGGTAGGTCCAATTCAG	$(TTTA)_3$	60
M28	GAAAGGGTCACGGATCATTCTA/AATCACCCAATAAGGAAGCTCA	$(AAAT)_3$	60
M29	CGTCTTCACTCACATGGTACATT/GTGCTTGCTTTGCTCTTCTGTA	$(AAAC)_3$	59
M30	TCTTTCTTGCGGAGAATGTTG/TATCAATAATCCCAATCGCTGG	$(GGAG)_3$	60
M31	AAAAGTAGGAGGCAAGGATTGA/CTAGCGAAGGTTCCCATGATAC	$(TTTA)_3$	59
M32	TGTCTTGTTTAGGCATTTGGTG/TACGAAGATTTCCCTTCTTTGC	$(TTTC)_3$	59
M33	GAAAGGGTCACGGATCATTCTA/AATCACCCAATAAGGAAGCTCA	$(AAAT)_3$	59
M34	GCAGAAGTCAAAGAAAAGCCAT/CTCAGCCCACACAATAGTAACG	$(TTTA)_3$	59
M35	AGCCATCACTTGGAATGGTAAT/TCAGAAAACGATAGATGCCCTT	$(AAAC)_3$	59
M36	TTCCCATGTTATACGTGCCATA/CCGCTGCTTATTATCCTTTCTG	$(TGTT)_3$	60
M37	CCTCCGGTACTTCACTTTATCG/CCAGGAGAAAAGCATCGAGT	$(GAAT)_3$	59
M38	CATTTGTGGTAATTGAGAGCCA/ACTGTGAAAAGTGTGAACCCCT	$(TGGT)_3$	59
M39	TATTCCACTGAGAGAGGTTTC/ CAAACACCAACTATTTCTGG	$(TTTA)_3$	53
M40	TCCGTTTGAGTTTGGTATTGGT/AGCAACCCTAATAACCCTCCAT	$(GTGA)_3$	60
M41	AGCAACTCATTTCTTCACCCTC/ATAGCTGTCAAGGTAGCCCATC	$(AATT)_3$	59
M42	TAGGCACTGAACCAAATCCAA/TCATCACTTTCTCAGCAGTCTCA	$(ATGA)_3$	60
M43	TTTTGTGTGTGACGTGGCTTAT/AAAGGTTTGTGGTGTAACCGTC	$(ATTT)_3$	60
M44	CTAACGGTGTTGAGTTTGACCA/AACATTGAAGCGAAATACCCTG	$(ATTC)_3$	60

M45	AACGAAGAGCAAACAGTAGGAA/TCGTAAACCCAGATGTCTTAGG	$(TTTA)_3$	58
M46	TGTCTTTGTTTCACCCCATAAC/ATGGTATGCCTCGTTAATCCA	$(TTAA)_3$	58
M47	TCAACTAGGGTGAGGAAAATCG/CCTAAAACGGTAAAACCCGAA	$(TTGT)_3$	60
M48	AAGGGAAGTAGAACGCATAGGA/ATTGAGACCCACGCTCTGAT	$(GCAT)_3$	59
M49	GCGAAATGAAAGGTGAAGAGAG/CCCCTCAATCTCAATCTCAATC	$(AAAT)_3$	59
M50	TATGGACAGGTATGGCCTTTTC/CTGGGGCTTAGATGATGAGTTT	$(TTTC)_3$	59
