# Supplementary material for: Identification and Characterization of Microsatellite Loci in Maqui (Aristotelia chilensis [Molina] Stunz) Using Next-Generation Sequencing (NGS)
Source: PLoS One. 2016 Jul 26;11(7):e0159825. doi: 10.1371/journal.pone.0159825 (PMC4961369; doi:10.1371/journal.pone.0159825)
Supplement: S4 Table — (PDF) [file pone.0159825.s004.pdf]

S4 Table. Frequency distribution of microsatellite loci of maqui (*A. chilensis*) by motif length

Motif length	N° of loci identified	Frecuency (%)	Mean repeats number
Di	5010	31.41	8.48
Tri	3843	24.09	4.91
Tetra	4078	25.57	3.22
Penta	1533	9.61	3.20
Hexa	1020	6.39	3.33
Hepta	378	2.37	3.30
Octa	88	0.55	3.28
