# Supplementary material for: Identification and Characterization of Microsatellite Loci in Maqui (Aristotelia chilensis [Molina] Stunz) Using Next-Generation Sequencing (NGS)
Source: PLoS One. 2016 Jul 26;11(7):e0159825. doi: 10.1371/journal.pone.0159825 (PMC4961369; doi:10.1371/journal.pone.0159825)
Supplement: S5 Table — (PDF) [file pone.0159825.s005.pdf]

S5 Table. Distribution of identified SSRs from maqui (*A. chilensis*) using MISA software according to SSR motif type and repeat number

**SSR motif type/Repeats number**	**3**	**4**	**5**	**6**	**7**	**8**	**9**	**>10**	**Total**
Di	NA	NA	NA	1715	977	703	428	1187	5010
Tri	NA	2249	762	387	201	108	53	83	3843
Tetra	3424	493	107	32	17	4	1	0	4078
Penta	1316	169	38	4	0	1	0	5	1533
Hexa	835	140	26	11	1	1	0	6	1020
Hepta	293	62	19	3	1	0	0	0	378
Octa	76	9	0	1	0	0	1	1	81

Na: not analyzed
