# Supplementary material for: Identification and Characterization of Microsatellite Loci in Maqui (Aristotelia chilensis [Molina] Stunz) Using Next-Generation Sequencing (NGS)
Source: PLoS One. 2016 Jul 26;11(7):e0159825. doi: 10.1371/journal.pone.0159825 (PMC4961369; doi:10.1371/journal.pone.0159825)
Supplement: S7 Table — (PDF) [file pone.0159825.s007.pdf]

S7 Table. Putative anthocyanidin-related genes found in partial sequences of maqui (*A. chilensis*) predicted by Blast2go software.

Sequence Name	Sequence Description	Sequence Length	Mean Similarity (%)
IYJ1KEP07HYLFD.g9198.t1	phenylalanine ammonia-lyase from *Populus nigra*	277	95.85
IYJ1KEP07H7GAU.g4318.t1	phenylalanine ammonia-lyase from *Pittosporum tobira*	474	98.15
IYJ1KEP07H6ZJF.g7092.t1	trans-cinnamate 4-monooxygenase from *Morus notabilis*	214	94.85
IYJ1KEP07IUH46.g7558.t1	csy4-coumarate: ligase-like from *Nicotiana sylvestris*	108	72.0
IYJ1KEP07H124X.g9021.t1	chalcone synthase from *Echinacea angustifolia*	258	67.4
IYJ1KEP07IJEH2.g2174.t1	chalcone synthase from *Betula pendula*	178	91.3
ConTiG00598.g376.t1	bifunctional dihydroflavonol 4-reductase flavanone 4-reductase from *Morus notabilis*	225	80.25
IYJ1KEP07IKYGY.g7.t1	rhamnose:anthocyanidin-3-glucoside rhamnosyltransferase from *Petunia axillaris*	443	80.05
IYJ1KEP07IO3M4.g435.t1	leucoanthocyanidin from *Ricinus communis*	405	71.2
IYJ1KEP07IFNXD.g934.t1	leucoanthocyanidin from *Ricinus communis*	411	63.95
IYJ1KEP07H6GPM.g1314.t1	basic helix-loop-helix dna-binding superfamily isoform 3 from *Theobroma cacao*	356	76.4
IYJ1KEP07IA2XV.g1895.t1	r2r3-myb transcription from *Ricinus communis*	215	98.9
IYJ1KEP07H8XUO.g7776.t1	r2r3 myb4b c2 repressor motif protein from *Vitis vinifera*	351	97.45
IYJ1KEP07H88FT.g673.t1	r2r3 myb4b c2 repressor motif protein from *Vitis vinifera*	426	90.85
